# Supplementary material for: Physics-embedded inverse analysis with algorithmic differentiation for the earth’s subsurface
Source: Sci Rep. 2023 Jan 13;13:718. doi: 10.1038/s41598-022-26898-1 (PMC9839692; doi:10.1038/s41598-022-26898-1)
Supplement: Supplementary file 1 — Supplementary Information. [file 41598_2022_26898_MOESM1_ESM.pdf]

Supporting information for "Physics-embedded inverse analysis with algorithmic differentiation for the earth's subsurface"

Hao Wu[1,*], Sarah Y. Greer[1,2] and Daniel O'Malley[1]

[1]Computational Earth Science, Los Alamos National Laboratory, Los Alamos, NM 87545

[2]Massachusetts Institute of Technology, Cambridge, MA 02139

[*]corresponding author: wu_hao@lanl.go

December 14, 2022

Contents

1. Figure S1: The convergence of the inverse analysis is shown for different values of nz for Gaussian fields.

2. Figure S2: The convergence of the inverse analysis is shown for different values of nz for bimodal fields.

3. Figure S3: The convergence of the inverse analysis is shown for different values of nz for hydraulic fracture fields.

4. Figure S4: The convergence of the inverse analysis is shown for different values of nz for seismic inversion.

5. Figure S5: The comparison between the observational data and outputs of the forward mode for bimodal fields.

6. Figure S6: The comparison between the observational data and outputs of the forward mode for hydraulic fracture fields.

7. Figure S7: The comparison between the observational data and outputs of the forward mode for seismic inversion.

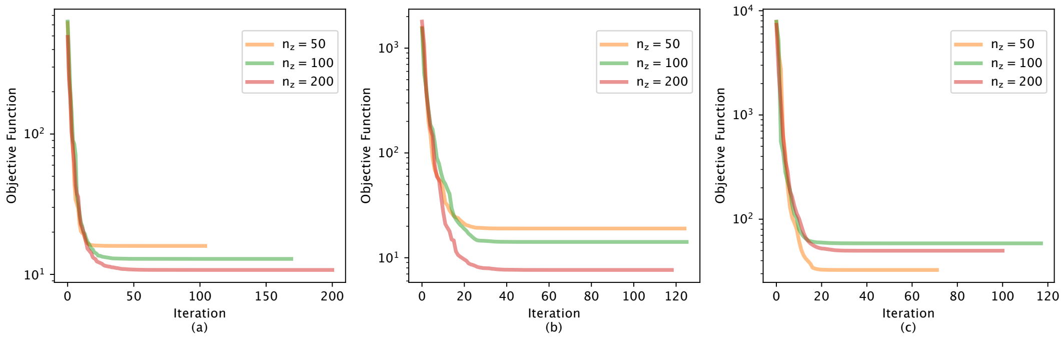

Figure S1

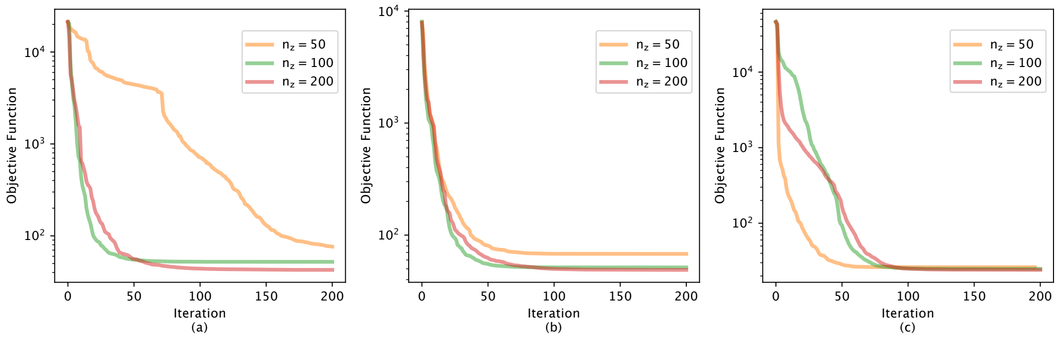

Figure S2

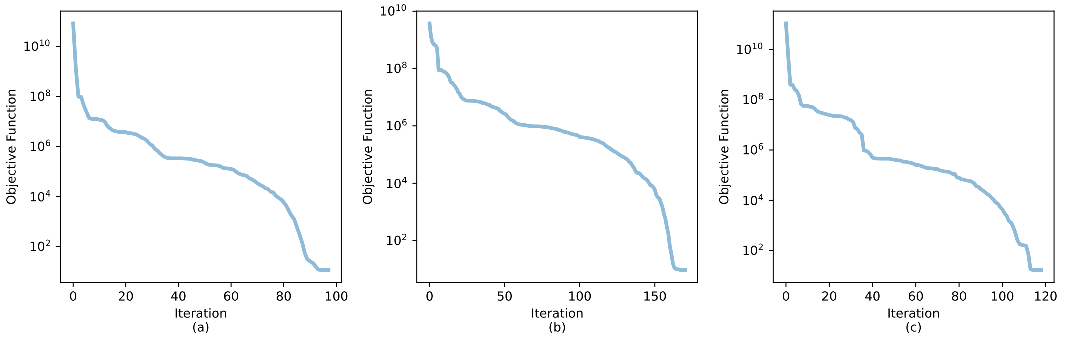

Figure S3

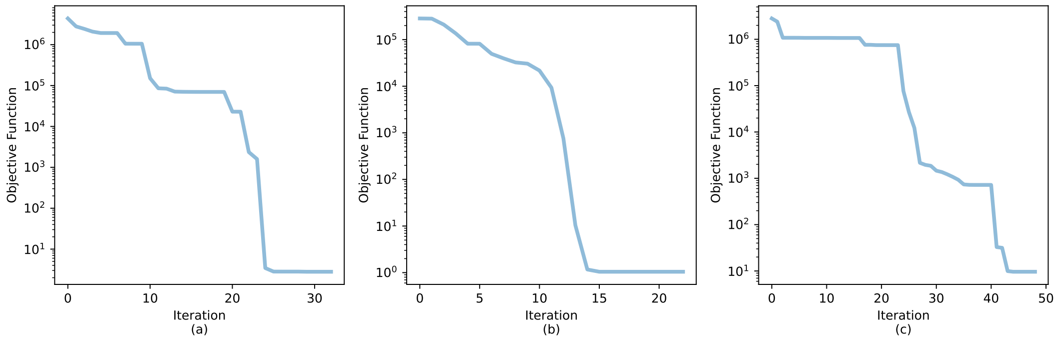

Figure S4

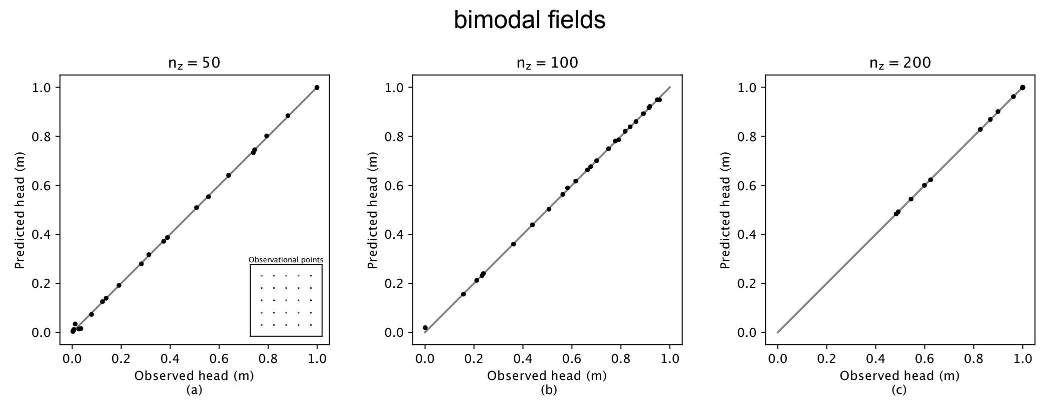

Figure S5

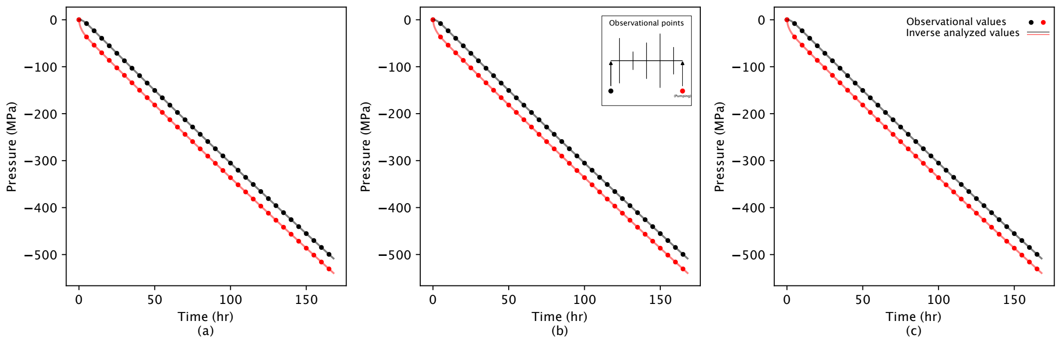

Figure S6

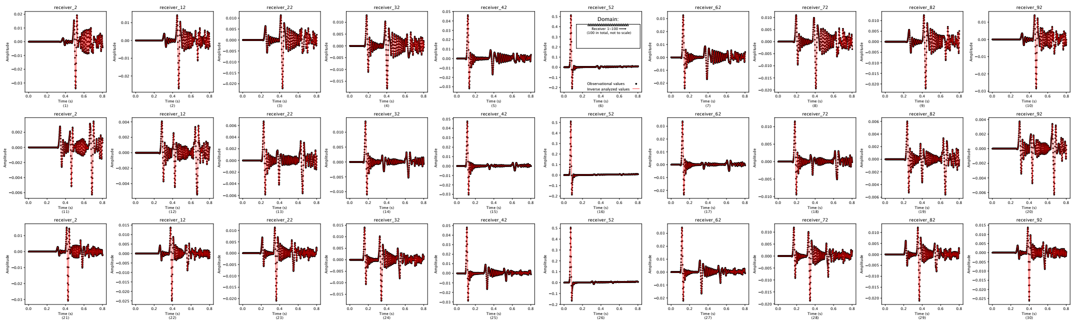

Figure S7
